# A literature review analysing endorsed performance and quality-in-care measures for emergency department assessment

**DOI:** 10.1186/1757-7241-21-S2-A12

**Published:** 2013-09-09

**Authors:** Christian Michel Sørup, Peter Jacobsen, Jakob Lundager Forberg

**Affiliations:** 1 Management Engineering, Technical University of Denmark, Denmark; 2 The Emergency Department, Hillerød Hospital, Denmark

## Background

Evaluation of the performance of an emergency department (ED) remains a difficult task due to the lack of consensus on performance measures that reflects both high quality and efficiency. Hence, this study describes, maps, and critically evaluates what performance measures that the published literature regards as being most relevant in assessing overall ED performance.

## Methods

A systematic literature review in the databases of PubMed, Cochrane Library, and Web of Science of articles on suggested ED performance measures.

## Results

A number of articles addressed this study’s objective (n = 14 of 46 unique hits). Time intervals and patient-related measures were dominant in the recommendations made in studies from US, UK, Sweden and Canada. Length of stay (LOS), time between patient arrival to initial clinical assessment, and time between patient arrivals to admission are recommended by the majority of studies. Concurrently, ‘patients left without being seen’ (LWBS), unplanned re-attendance within a maximum of 72 hours, mortality/morbidity, and number of unintended incidents make out the most recommended performance measures related directly to the patient. Performance measures related directly to employees were only stated in two of the 14 included studies. Operational performance measures are deemed covered for by the two clusters 1) time intervals and 2) patient-related performance measures.

## Conclusion

54 performance measures have been extracted from 14 studies. ED time intervals are the most recommended performance measures followed by patient centeredness and safety performance measures. ED employee related performance measures are rarely mentioned in the investigated literature. Further work will include working towards consensus agreement on ED performance measures that preferably should include several aspects of performance. Moreover, investigation of the interconnectivity between the performance measures and how to measure if launched initiatives have the wanted effects.

